# News and Gossip

**Published:** 1870-10

**Authors:** 


***Communications***

***News and Gossip.***

Profs. Huxley and Tyndall propose to visit and lecture in this country, sometime the ensuing year. The former gentleman's official position in the British School of Mines yields him about $3,000 *per annum*, a sum which would be vastly surpassed by a series of lectures by him, anywhere in the United States.——Prof. A. Winchell, State Geologist of Michigan, presented, at the recent meeting of the American Association for the advancement of Science, a paper on the Magnetic Wells of Michigan. We hope to have more light on the subject when it is printed.——An enterprising citizen of Chicago, claims to have discovered a magnetic water on his farm a few miles out of the city. Prof. Winchell of Ann Arbor, and Dr. Duffield of Detroit, are quoted in the connection. Does anybody recollect Prof. Silliman's (the younger) elaborate pamphlet on the petroleum lakes of California? *Vive la bagatelle!* Now let us have Chicago River Water duly bottled, certified by the Michigan State Geologist, assisted by the Chemist thereof, and sold as NATIVE MEDICATED SOUP. The primeval forms are there, and artificial combinations as complicated as the most fashionable mixtures of heterogeneous incongruities that adorn the pages of our apothecaries' prescription books. As the old Hopkins saw-mill at Grand Haven, Michigan, has been reconstructed to a universal sanitarium and double-back-action rejuvenator, so let us have the Cook Co. and Mercy castles of *ochlesis* and disease, converted into water cures, (with magnetism and soup thrown in,) connecting each with the North Branch, or Healey's slough, and the recent *tap* of the Styx, "down by the Riverside." Stamps to be furnished by the inmates.——The Woman's College is *fait accompli*, and our table is covered with communications on the subject. We must decline all alike, or fill two or three numbers of the JOURNAL. We have only to explain that we do *not* know why all the Faculty, but one, are nominally male; nor why the Professor of Diseases of Children, is an inveterate old bachelor. That is their business, and we are no more called upon to explain it than why a so-called male college receives students for one session by promising each of them professional chairs the next. We repeat, it is not our business, as journalists, to answer such questions. Unquestionably, notes addressed to the Secretaries of the respective faculties (enclosing stamps for return postage) would elicit prompt replies. The advocates of Mesdames G——s, L—v—y, and B—f—t, must not ask us to take sides.——Scribonius Largus mentions a black torpedo applied alive to the head as a remedy for headache. Several other ancient authors confirm the efficacy of the practice. Thus it will be seen the *gymnotus* antedated modern galvanism.——Hennig asserts that catarrh of the Fallopian tubes, is more common than similar affections of the body, neck or vagina. Now then, let the endoscope be fastened on to the end of an improved speculum, the cervix be duly stretched and the enemy be dislodged by the right or left flank movement of the *porte caustique.*——Vegetable charcoal is recommended as a local application to burns. In young children a tepid bath containing a little sulphate of iron in solution, is used for the same purpose at the hospital at Lausanne.——Dr. Davis, of Iowa, claims that Bromide of Ammonium is *innervating*, in striking contrast to Bromide of Potassium which is *enervating.*——The *Edinburg Journal* says, that Balsam of Peru is *the* remedy for scabies. The patient is stripped and well and carefully rubbed over with the balsam from crown to heels, avoiding abrasions. It soaks into galleries and eggs, and kills everything on the body in an hour. A second rubbing ten days after, kills any stray animalcule or products of eggs that may have been in the clothes the first time. After this the clothes need no disinfection of any sort.——The *Imparziale*, contains (*Nashville Journal*) a vigorous attack upon the practice of circumcision, by Dr. Sonsino, who claims that as a hygienic measure it is a failure; that the principles of medical ethics do not allow of its performance as a prophylactic of venereal affection; and that as a religious rite, it is deserving of condemnation. On the point of prophylaxis, we remark that last month we had occasion to treat a chancre near the frænum of an adult circumcised in infancy. Our observation leads us to believe such cases extremely rare.——An exclusively skim milk diet is said to be not only useful in diabetes, Addison's disease, Bright's disease, but in the treatment of corpulency to be far more efficacious than Bantingism.——A London telegram says that 150 persons are dying, daily, of cholera, in St. Petersburgh. Which reminds us of a practice suggested to us several years since by our valued friend T. O. Edwards, M.D., now of Lancaster, Ohio. On the approach or occurrence of collapse use this:

| | | |
|---|---|---|
| ℞ | Strychniæ Chrys., - - - - - - | gr. ss; |
| | Sp. Terebinth., - - - - - - | ʒ ij; |
| | Muc. Gum Acac., - - - - - - | ℥ viij; |

M. Intim.

S. A tablespoonful every fifteen minutes; shake well.

This will be found useful in many other grave forms of collapse.

Germane to Prof. Hodgen's discovery, we are reminded of this scissoring:

***A Hard Customer.***

The scene of the following incident was a Utica restaurant. A man recently entered the place and ordered a very elaborate dinner. He lingered long at the table, and finally wound up with a bottle of wine. Then lighting a cigar he had ordered, he leisurely sauntered up to the counter and said to the proprietor: "Very fine dinner, landlord; just charge it to me, I haven't got a cent." "But I don't know you," said the proprietor, indignantly. "Of course you don't. If you had you wouldn't have let me had the dinner." "Pay me for the dinner, I say." "And I say I can't." "I'll see about that," said the proprietor, who snatching a revolver out of a drawer, leaped over the counter and collared the man, exclaiming, as he pointed it at his head, "Now see if you'll get away with that dinner without paying for it, you scoundrel." "What is that you hold in your hand?" said the impecunious customer, drawing back. "That, sir, is a revolver, sir." "Oh, that's a revolver, is it? I don't care a damn for a revolver, I thought it was a stomach pump."

Morphine dissolved in the proportion of one part of the hydrochlorate to thirty of flexible collodion, is advised as a local application with a camel's hair brush, in neuralgia.——Owing to the neglect of vaccination, small pox is on the increase in London. ——Dr. Charvet extols the use of a solution of permanganate of potash in water, in fœtid expectoration.——The *Medical Press and Circular* says, that a combination of strychnia and iodide of ammonium is much to be lauded in fatty degeneration of the heart. The thirtieth of a grain of strychnia, gradually increased to the tenth, is to be given with two grains of the iodide of ammonium, in a little spirits of chloroform and camphor julep, as a vehicle. In some instances the iodide was rubbed over the cardiac region in the form of a cerate. Of course, other therapeutic measures may be indicated.——A Kentucky Medical College, somewhat noted for its advocacy of high fees, has resorted to the old dodge of instituting a Beneficiary Scholarship for each Congressional district in the Southern States. A religious contemporary, which evidently has not yet cut its eye teeth, commends this as a *noble* benefice. In *Jahr's Manual* we read that small doses of gold produce excessive scruples of conscience. We fear that the *large* fees of the college with which our friend Gaillard is connected has had a contrary effect. However, we are assured "that with proper and welcome delicacy, the names of those who have secured the Beneficiary Scholarships will be known only to the Dean of the Faculty." This is what we may call "ratting to the opposition."

*Pro pudor!* This attempt to bolster up a feeble school, reminds us of the old examination and its result:

Ques. *Quod est creare?*

Ans. *Creare est facere aliquid ex nihil.*

R. *Ergo, Creamus te Doctorem.*

——Sidney Smith said that "Whoever discourses in the accusative case, must order his language with great care and precision," an injunction our New York weekly contemporary overlooks or disregards. In his attack upon the American system, he committed simply a blunder. When his attention is directed to plain facts, he is anxious to drop the subject. Under cover of a pun (somewhat too obvious to be bad enough for reprehension, as "facetious") he disappears from the arena of argument. But trivial as is the pun, we accept it in all its length, breadth and thickness. We immensely prefer the Russian (*Rush*-ian says our friend) system to the Chinese. Russia has shown a spirit of progress and illustrated a real advancement, within the century past, that it will take ages for China to parallel.——Why does not somebody manufacture a hypodermic syringe that will not get out of order, every third time trying? The cheap, badly constructed toys of the kind we have seen, and have been occasionally obliged to use, are a disgrace to the craft. A similar remark may be made with regard to the atomizing apparatus. This is frequently a useful affair, but, so far, as primitive in its construction as a wooden plough. The brass and glass in it do not relieve it from its absurdity. Let us have something better.——Correspondents of the *American Journal of Pharmacy* commend the following combinations as preferable to the officinal *Mistura Cretæ:*

℞ Cretæ Preparat.,
Pulv. Gm. Acaciæ,
Glycerin, - - - - - āā ℥ j;
Aq. Cinnamomi, - - - ℥ xv;
M.

℞ Cret. Preparat., - - - - - ℥ ss;

P. Sacch. Alb.,

P. Gm Acaciæ, - - - - āā ʒ ij;

M. S. artem.

A dram of this powder to half an ounce each of water and cinnamon water, for each fluid ounce of chalk mixture required.

Dr. T. S. Dowse gives in the *London Pharm. Journal* (*Am. Jour. Ph.*) the following as the best formula for the preparation of Chlorodyne. He does not give the dose, but it is probably the same as that of the original. It does not contract the pupil:

| | | |
|---|---|---|
| ℞ | Belladonnæ Ext. | ʒij; |
| | Morphiæ Muriatis, | gr. xxx; |
| | Ætheris Rectif., | f ʒ viij; |
| | Chloroformi, | f ʒ viij; |
| | Acid Hydrocyanic dilut., | f ʒ iv; |
| | Ol. Menth. Piperit., | gtt. xxx; |
| | Capsicine, | gr. vj; |
| | Mist. Acaciæ, | ʒ xx; |
| | Caramel, | ʒ j; |
| | Theriacæ, | ad ʒ lx; |

M. S. artem.

The *American Practitioner* gives a different formula, which presents some advantages:

| | | |
|---|---|---|
| ℞ | Chloroformi, | ℥ vj; |
| | Tinct. Capsici, | ʒ vj; |
| | Tinct. Cannabis, | ℥ iss; |
| | Ol. Menth. Pip., | gtt. xxxvj; |
| | Morph. Hydrochlor., | gr. lx; |
| | Acid. Hydrocyan. (Schule's), | ʒ j; |

M.

Dr. Metcalfe recommends in *Diphtheria:*

| | | |
|---|---|---|
| ℞ | Strychniæ, | gr. j; |
| | Acid Nitric, Dil., | ʒ j; |
| | Aq. Pur., | ʒ vij; |

M. S. Three to five drops three or more times a day, to a child three years old.

The following is recommended as a local and general antiseptic in similar affections:

| | | |
|---|---|---|
| ℞ | Iodinii Bromid., | gtt. xx; |
| | Syrup. Acaciæ, | ℥ iv; |

M. S. Apply locally and give internally a teaspoonful every four hours.

Dr. George Bayles commended the following, in obstinate vomiting:

| | | |
|---|---|---|
| ℞ | Chloroform., | ℥ ij; |
| | Tinct. Aconiti, | ʒ iss; |
| | Tinct. Opi. Camph., | ℥ ss; |
| | Aq. Pur., | ℥ iij; |

M. S. A teaspoonful every hour, p. r. n.

An old mixture, favorite in the Montreal General Hospital, is the following:

℞ Potassæ Nitrat., - - - - ℥ ss;
Acet. Scillæ, - - - - - ℥ iv;
Tr. Opi. Camph., - - - - ℥ iv;
Antim. Pot. Tart., - - - - - gr. xij;
Aq., - - - - - - O iiiss;
M. S. A tablespoonful, in febrile and inflammatory affections requiring sedative and eliminant action.

Dr. J. B. Chagnon, in the *Canada Med. Journal*, commends as infallible, for after pains:

℞ Acid Citric, - - - - - gr. v;
Aq., - - - - - - ℥ ij;
M. ft. haustus.
S. To be taken at once and repeat, p. r. n. every five hours in troublesome after-pains.

He says it acts as a nervine and as a preventive of inflammation.

Dr. Garretson of the University Hospital, according to the *Philadelphia Reporter*, has discovered a "specific for erysipelas." Here it is:

℞ Tinct. Ferri Chlorid.,

Tinct. Cinchonæ, - - - āā f ʒ ij;

Quiniæ Sulph., - - - - gr. xxx;

Aq., - - - - - - f ℥ iss;

M. S. Apply with a brush, four times a day.

The learned doctor, in a recent clinic, intimated that "common sense" taught confidence in specifics. [*Omne ignotum pro magnifico.*]

Dr. A. Given (*Examiner*) commends the following *novel* mixture in typho-enteric fever:

℞ Ol. Terebinth., - - - - ʒ ij;
Pulv. Acaciæ,
Sacch. Alb., - - - - - āā ʒ iij;
Aq. Menth., - - - - - ℥ ij;
Tinct. Opi., - - - - - ʒ ij;
M. S. A teaspoonful every two or four hours until the bowels are checked; then lengthen out the dose to intervals of every six or eight hours, until tenderness has subsided.

"The turpentine penetrates the ulcerated surfaces, corrects the morbid action, stimulates the tissues, and sets up the healing process. The tincture of opium is beneficial in arresting the peristaltic movement of the bowels, and allaying the morbid irritability of the parts; and by its power of arresting secretion, it promotes resolution." Regin Thompson, the fever man, prefers Oil of Sassafras and Soda. *Suum cuique*, etc.——A somewhat older writer, Paulus Egineta, in treating of urinary calculi, commends a species of Swallow, thus: "When this bird is pickled whole, and frequently eaten in a raw state, it makes the stones which are already formed be passed with the urine, and prevents them from being formed again, and if it be burned alive, entire, with its wings, and if the ashes by itself and along with pepper and a moderate quantity of Indian leaf, be drunk out of mulse, it will do the same thing."

